# Factors Associated With the Decay of Anti-SARS-CoV-2 S1 IgG Antibodies Among Recipients of an Adenoviral Vector-Based AZD1222 and a Whole-Virion Inactivated BBV152 Vaccine

**DOI:** 10.3389/fmed.2022.887974

**Published:** 2022-06-13

**Authors:** Sivaprakasam T. Selvavinayagam, Yean Kong Yong, Hong Yien Tan, Ying Zhang, Gurunathan Subramanian, Manivannan Rajeshkumar, Kalaivani Vasudevan, Priyanka Jayapal, Krishnasamy Narayanasamy, Dinesh Ramesh, Sampath Palani, Marie Larsson, Esaki M. Shankar, Sivadoss Raju

**Affiliations:** ^1^State Public Health Laboratory, Directorate of Public Health and Preventive Medicine, Chennai, India; ^2^Laboratory Center, Xiamen University Malaysia, Sepang, Malaysia; ^3^School of Traditional Chinese Medicine, Xiamen University Malaysia, Sepang, Malaysia; ^4^Chemical Engineering, Xiamen University Malaysia, Sepang, Malaysia; ^5^Government Corona Hospital, Chennai, India; ^6^Department of Biomedicine and Clinical Sciences, Linkoping University, Linköping, Sweden; ^7^Infection Biology, Department of Life Sciences, Central University of Tamil Nadu, Thiruvarur, India

**Keywords:** AZD1222, BBV152, COVID-19, IgG decay, SARS-CoV-2, vaccination

## Abstract

**Background:**

The magnitude of protection conferred following recovery from COVID-19 or by vaccine administration, and the duration of protective immunity developed, remains ambiguous.

**Methods:**

We investigated the factors associated with anti-SARS-CoV-2 S1 IgG decay in 519 individuals who recovered from COVID-19 illness or received COVID-19 vaccination with two commercial vaccines, viz., an adenoviral vector-based (AZD1222) and a whole-virion-based inactivated (BBV152) vaccine in Chennai, India from March to December 2021. Blood samples collected during regular follow-up post-infection/-vaccination were examined for anti-SARS-CoV-2 S1 IgG by a commercial automated chemiluminescent immunoassay (CLIA).

**Results:**

Age and underlying comorbidities were the two variables that were independently associated with the development of a breakthrough infection. Individuals who were >60 years of age with underlying comorbid conditions (viz., hypertension, diabetes mellitus and cardiovascular disease) had a ~15 times and ~10 times greater odds for developing a breakthrough infection and hospitalization, respectively. The time elapsed since the first booster dose was associated with attrition in anti-SARS-CoV-2 IgG, where each month passed was associated with an ebb in the anti-SARS-CoV-2 IgG antibody levels by a coefficient of −6 units.

**Conclusions:**

Our findings advocate that the elderly with underlying comorbidities be administered with appropriate number of booster doses with AZD1222 and BBV152 against COVID-19.

## Background

The COVID-19 pandemic has caused an unprecedented global crisis, and having lasted for more than 2 years, has resulted in over 524 million COVID-19 cases, claiming >6.27 million deaths by Mid-May 2022, causing huge levels of economic damage (1, 2). Although antiviral agents against SARS-CoV-2, the virus causing COVID-19, are becoming available, vaccines and public health interventions remain the most promising approach against this global peril. Notwithstanding anti-SARS-CoV-2 vaccines are successful in reducing the mortality and morbidity rates, and the level of neutralizing antibodies has correlated with protection against SARS-CoV-2 reinfection (3) as well as severe COVID-19 (4–6), breakthrough infections do continue to recur. Importantly, it still remains a conundrum, how long this vaccine-induced acquired immunity would last in an individual.

Several studies have investigated the dynamics and the duration of protection of neutralizing antibodies developed following the onset of natural SARS-CoV-2 infection or vaccination (7–11). The results thus far remain inconsistent, with some reporting rapid waning of antibodies months after virus exposure or following vaccine administration (7–13), whereas others report the prolonged presence of neutralizing antibodies (14–16). This discrepancy partly stems from numerous factors including differences in patient demography, vaccine type, and number of infections/vaccinations one has become exposed to, SARS-CoV-2 strains, intrinsic properties of immunity, underlying disease and many other miscellaneous factors. Given the complex interaction between intrinsic and extrinsic factors, the duration of protective immunity developed post-vaccination against COVID-19 still remains uncertain. Here, by using an appropriate statistical model, we investigated the factors such as age, gender, vaccine status, type of vaccine viz., a viral vector-based AZD1222 and an inactivated BBV152 vaccine as well as comorbidities and their association with antibody decay.

## Materials and Methods

### Study Population

We conducted a population-based study among Chennai's adult who received treatment for COVID-19-related illness or received COVID-19 vaccination at the State Public Health laboratory, Directorate of Public Health and Preventive Medicine, Chennai, India, and the Government Corona Hospital, Chennai, India from March until December 2021. The inclusion criteria were that the participants needed to be >18 years, and there were no exclusion criteria. The medical records of the participants were reviewed and data such as patient demography, comorbidities, history of SARS-CoV-2 infection, date and type of vaccine received were recorded. Blood samples were collected during regular follow-up post-infection and/or post-vaccination, and tested for their levels of anti-SARS-CoV-2 IgG. The study was approved by the Human Ethics Committee of the Madras Medical College (EC No. 03092021).

### Diagnosis of COVID-19 and Breakthrough SARS-CoV-2 Infection

Diagnosis of COVID-19 was made based on clinical and laboratory diagnoses; the former based universal Clinical Criteria 2021 defined by the Centers for Disease Control and Prevention, Atlanta, USA (https://ndc.services.cdc.gov/case-definitions/coronavirus-disease-2019–2021/), and the later confirmed by a commercial TaqPath^TM^ COVID-19 RT-PCR test (Applied Biosystems, Thermo Fisher Scientific, Pleasanton, CA) for the *in vitro* qualitative detection of nucleic acid from the SARS-CoV-2. Breakthrough cases were detected after tele-consultation with the vaccinated individuals following the development of COVID-19 symptoms and a laboratory diagnosis with the aforesaid RT-PCR test.

### Anti-SARS-CoV-2 S1 IgG Chemiluminescent Assay

Blood collected were tested for their levels of anti-SARS-CoV-2 S1 IgG by VITROS anti-SARS-CoV-2 S1 IgG assay, a commercial automated chemiluminescent immunoassay (CLIA), according to manufacturer's instructions using a VITROS Anti-SARS-CoV-2 IgG Calibrator on the VITROS ECi/ECiQ/3600 Immunodiagnostic Systems and the VITROS 5600/XT 7600 Integrated Systems. The assay targeted to the spike protein S1 antigen and the cut-off (minimum detection limit) was ≥1.00.

### Statistical Analyses

The primary analysis was to compare individuals with natural infection with those who received the AZD1222 and the BBV152 vaccines. Comparison of categorical variables was tested using the Chi-Square test, whereas continuous variables (e.g., age) were compared using the unpaired *t*-test. Potential risk factors for breakthrough infection and hospitalization such as demographics between those who had natural infection, or received AZD1222 and BBV152 vaccination were evaluated by simple and adjusted binary logistic regression. The odds ratio (OR) and 95% confidence interval (CI) were estimated. The predictive power of age in predicting a breakthrough infection and hospitalization were examined using the receiver operating characteristic (ROC) analysis. The decay of anti-SARS-CoV-2 S1 antigen IgG levels was assessed using an adjusted linear regression. Statistical analyses were performed using PRISM, version 5.02 (GraphPad Software, San Diego, CA). Binary regression was performed using SPSS, version 20 (IBM, Armonk, NY), Two-tailed P <0.05 was considered as statistical significance for all the tests performed, and *P*-values <0.05, <0.01, <0.001, <0.0001 were marked as ^*^, ^**^, ^***^ and ^****^, respectively.

## Results

### Demographics and Cohort Characteristics

**Figure 1:**
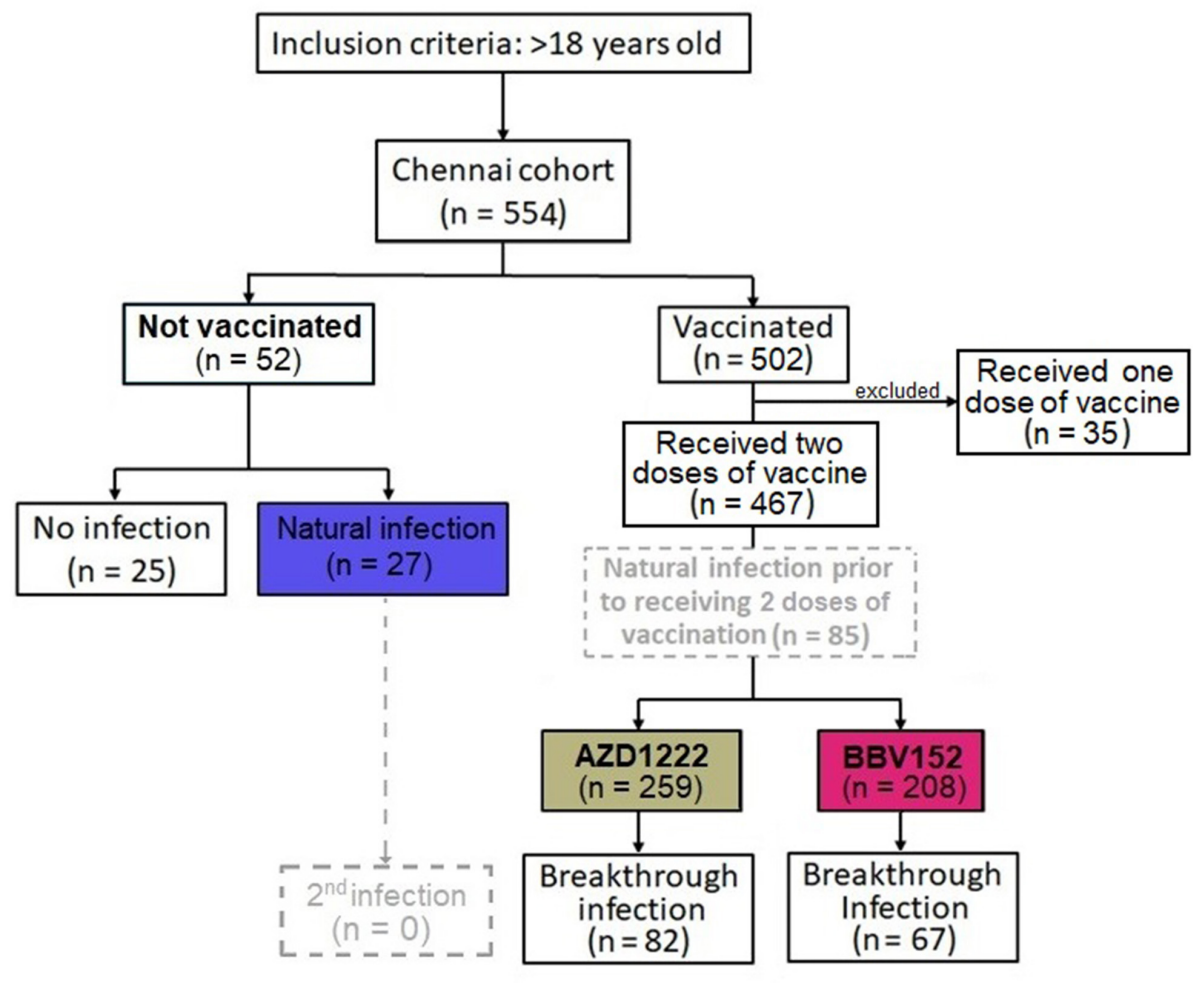
Flow diagram of 519 participants recruited into the Chennai (India) Cohort from March to December 2021. Based on the sequence of natural infection and type of vaccine administered, the cohort is divided into four groups viz., **(i)** No vaccination, no natural infection, **(ii)** no vaccination, with natural infection, **(iii)** vaccination with AZD1222 and **(iv)** with BBV152. Of note, a small fraction of vaccine recipients had a documented history of natural infection prior to receipt of the corresponding vaccine (*n* = 85); whilst another group of participants developed a breakthrough infection 14 days post-vaccination (*n* = 149). The colored boxes, present three main patient groups in the investigation.

In India, two vaccines were initially approved for administration to the public, one the adenoviral vector vaccine AZD1222 (ChAdOx1) manufactured by the Serum Institute of India, Pune, and the other, a whole-virion inactivated BBV152 vaccine developed by Bharat Biotech International Limited, Hyderabad, in collaboration with the Indian Council of Medical Research (ICMR) (17). Five-hundred and fifty four individuals recruited into the study were separated into two groups, i.e., “unvaccinated” (*n* = 52) vs “vaccinated” (*n* = 502). The “unvaccinated” group was further bifurcated into two sub-groups with those who did not have a history of natural infection of SARS-CoV-2 (*n* = 25) and those who had a natural COVID-19 infection (*n* = 27). Those who received only one dose of vaccine were excluded from analysis (*n* = 35). The vaccinated group was also divided into two groups i.e., participants who received the AZD1222 (*n* = 259) and those who received the BBV152 (*n* = 208) vaccines. Of note, a small fraction of vaccinees had developed a natural infection prior to completion of two doses of vaccination (*n* = 85); whilst another portion of the participants had developed a breakthrough infection after completion of two doses of vaccination (*n* = 149). The colored boxes in Figure 1 represent the three main study groups in our investigation. The median age of the cohort was 34 years with an interquartile range (IQR) of 26–52, with 47.8% male participants. Of note, 14.3% (*n* = 74) of the participants had some form of underlying comorbid conditions such as hypertension (*n* = 37), diabetes mellitus (*n* = 24), and heart disease (*n* = 4). Of all the participants, 176 (33.9%) were infected by SARS-CoV-2 (Table 1). There was a significantly higher number of individuals with COVID-19 among the non-vaccinees (51.9%) as compared to the vaccine recipients (AZD1222 = 31.7% and BBV152 = 32%) (*P* = 0.016). There was no significant difference between the percentage of individuals developing breakthrough infection with both the vaccines, indicating that the protective efficacy of both the vaccines are similar and the onset of a breakthrough infection appears to have been attributed to inadequate cross-neutralizing potential conferred by the vaccine to the circulating virus.

**Table 1:** Clinico-demographic characteristics of the Chennai, India study cohort.

**Characteristics**	**Total**	**Unvaccinated**	**Vaccinated (*****n*** **=** **467)**		***P*** **-value**	
			**AZD1222**	**BBV152**	**a**.	**b**.
**Number of participants, no. (%)**	519 (100)	52 (10)	259 (55.5)	208 (44.5)	…	…
**Age, year; median (IQR)**	34 (26-52)	32 (25.5–42.7)	35 (27-53)	34 (25-52)	0.419	
**Gender, (male) no, (%)**	248 (47.8)	27 (51.9)	115 (44.4)	106 (51)	0.855	
**Healthcare workers, no. (%)**	237 (45.7)	18 (34.6)	117 (45.2)	102 (49)	0.097	
**Comorbidities, no. (%)**	74 (14.3)	7 (13.5)	36 (13.9)	31 (14.9)	0.775	
*Hypertension*, no. (%)	37 (7.1)	4	19	14	…	
*Diabetes*, no. (%)	24 (4.6)	2	10	12	…	
*Heart disease*, no. (%)	4 (0.8)	0	3	1	…	
*Others*^†^, no. (%)	9 (1.7)	1	4	4	…	
**SARS-CoV-2 infection; no. (%)**	176 (33.9)	27 (51.9)	82 (31.7)	67 (32)	0.016^a*^	0.25

*Others^†^ consist of asthma = 4, arthritis = 2, cancer/cured cancer = 2, chronic lung disease = 1. (a) Comparison between three groups, i.e., Unvaccinated, AZD1222 and BBV152; (b) Comparison between, AZD1222 and BBV152*.

* *P < 0.05*.

### Factors Predisposing the Development of a Breakthrough Infection

**Figure 2:**
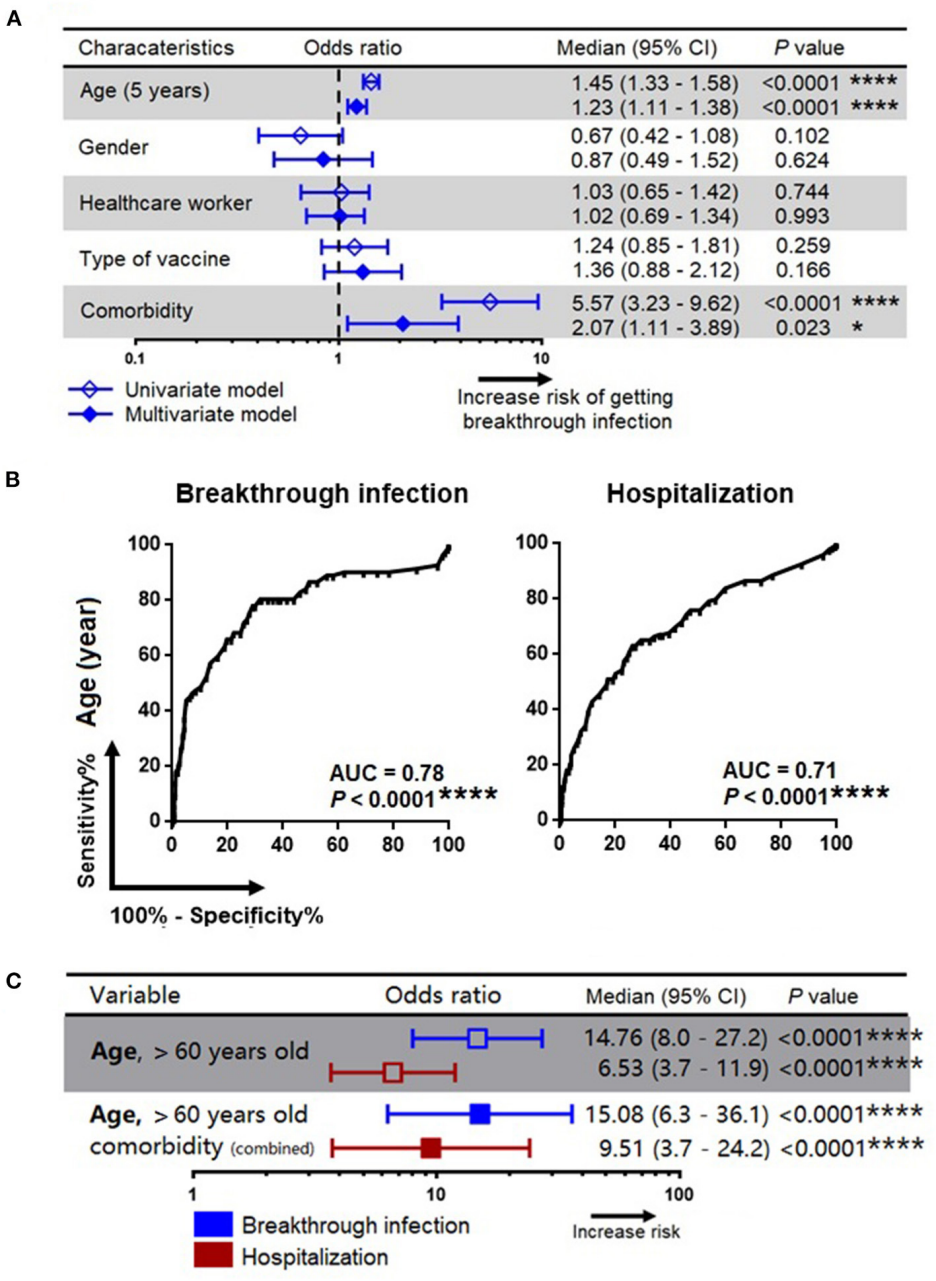
Association of patients' characteristics with risk for development of breakthrough infection and hospitalization. **(A)** A simple and adjusted binary regression models assessing the factors that associated with breakthrough infection. Odds ratios for values below or above threshold levels were displayed in a forest plot; median and 95% CI were calculated. **(B)** Receiver operating characteristics analysis for prediction of breakthrough infection and hospitalization. **(C)** Association of age and comorbidity with the risk of breakthrough infection and hospitalization. CI, confidence interval; Comorbidity refers to diabetes mellitus, cardiovascular disease, hypertension and other underlying medical conditions as detailed in Table 1; *, **, ***, *** represent *P* < 0.05, <0.01, <0.001, <0.0001, respectively.

Since a substantial proportion of individuals develop breakthrough infections despite administration with two doses of the vaccine, we sought to investigate the factors that predispose to breakthrough infection. The association between development of a breakthrough infection and other demographic parameters such as age, gender, occupation as healthcare workers, type of vaccine received and comorbidities were first assessed univariately using a binary regression model. Variables with *P*-value <0.05 will then be included in the multivariate analysis. Variables with *P*-value <0.05 were considered as independent predictors. Our multivariate analysis showed that age and underlying comorbidities were the two variables that were independently associated with development of a breakthrough infection. We also found that every increase in age by 5 years was associated with an increased risk of developing a breakthrough infection by 1.23 unit (95% CI=1.11–1.38; *P* < 0.0001). Further, an existing comorbid condition was associated with an increased risk of contracting a breakthrough infection by 2.07 units (95% CI=1.11–3.89; *P* < 0.023) (Figure 2A). We also found that age and comorbidity were independently associated with hospitalization (Table 2).

**Table 2:** Clinical and demographic characteristics associated with hospitalization.

**Characteristics**		**Univariate model**			**Multivariate model**	
	**Coeff**.	**(95% CI)**	***P*-value**	**Coeff**.	**(95% CI)**	***P*-value**
Age (5 years)	1.312	(1.224–1.401)	<0.0001 ^****^	1.267	(1.179–1.362)	<0.0001 ^****^
Gender	1.451	(0.988–2.128)	0.058	0.771	(0.509–1.168)	0.219
Vaccine status	0.653	(0.326–1.31)	0.229	…	…	…
Vaccine type						
AZD1222	0.687	(0.331–1.425)	0.313	…	…	…
BBV152	1.095	(0.732–1.637)	0.659	…	…	…
Comorbidity	3.973	(2.392–6.601)	<0.0001 ^****^	1.930	(1.098–3.394)	0.022 ^*^

Coeff, coefficient; CI, confident interval. ^*^, ^**^, ^***^, ^****^, represent P < 0.05, <0.01, <0.001, <0.0001, respectively.

The ROC analysis revealed that age was a strong predictor for the development of a breakthrough infection (area under curve, AUC=0.78; *P* < 0.0001) as well as hospitalization (AUC=0.71; *P* < 0.0001) (Figure 2B), and the cut-off age was determined as 60 years. Our binary regression analysis showed that participants who were >60 years of age and with underlying comorbid conditions had a ~15 times and ~10 times greater odds for developing a breakthrough infection and hospitalization, respectively (Figure 2C).

### Factors Associated With the Decay of Anti-SARS-CoV-2 S1 IgG

**Figure 3:**
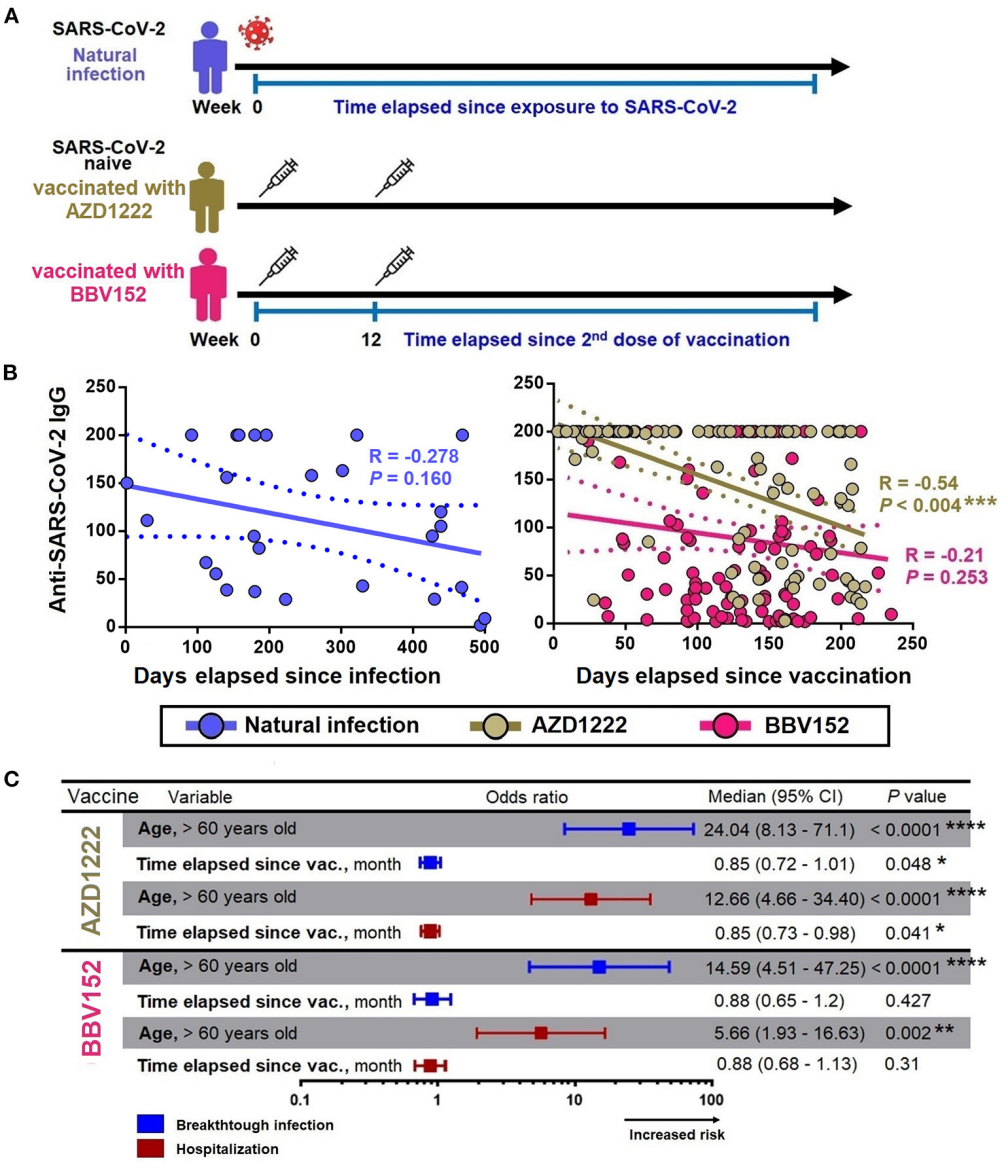
Factors associated with decay of anti-SARS-CoV-2 S1 IgG. **(A)** Anti-SARS-CoV-2 S1 IgG decay cohort design. **(B)** Spearman correlation between the levels of anti-SARS-CoV-2 S1 IgG with the time elapsed since exposure to infection/vaccine administration. **(C)** Binary regression models assessing the association between age (>60 years) and time elapsed since vaccination with breakthrough infection and hospitalization. Odds ratios were displayed in a forest plot; median and 95% CI were calculated. CI, confidence interval; month define as 30 days. *, **, ***, *** represent *P* < 0.05, <0.01, <0.001, <0.0001, respectively.

Given that the titer of antibodies will decay gradually with time, next we investigated the decay of anti-SARS-CoV-2 S1 IgG levels in individuals who experienced a natural infection, and having vaccinated with AZD1222 and BBV152. In this analysis, we only included those who had a natural infection (without vaccination, *n* = 27), and those who had received two doses of vaccination (*n* = 233). We excluded those who had natural infection prior completion of two doses of vaccination (*n* = 85) and those who had a breakthrough infection (*n* = 82 with AZD1222 and *n* = 67 from BBV152, total *n* = 149). The total number of participants in this analysis was (*n* = 260). Using a linear regression model, we first studied the decay of anti-SARS-CoV-2 S1 IgG levels across the two vaccine groups in comparison with natural infection, controlling for the time elapsed since recovering from a natural infection or after administration with the second dose of the respective vaccines (Figure 3A).

The univariate analysis showed that the anti-SARS-CoV-2 S1 IgG levels were waning progressively over time with varying rates, where the IgG levels in those who received AZD1222 were decayed slightly faster compared to that from a natural infection. Time lapse analysis showed that each month passed was associated with an antibody attrition levels by a coefficient of −6 unit (95% CI = −9.88 to −2.1; *P* = 0.003) and by a coefficient −5.22 unit (95% CI = −9.6 to −0.85; *P* = 0.019) in those who received AZD1222 and those who recovered from a natural infection, respectively (Table 3). In the adjusted model, we found that participants who were >60 years of age had an accelerated decay rate, where each month of lapse was associated with a decrease of IgG by a coefficient of 23 units (95% CI = −46.69 to −0.05; *P* = 0.047). However, such decay of IgG was only observed among participants who received AZD1222, but not among those who had a history of recovery from a natural SARS-CoV-2 infection (partly owing to a small sample size) and those received BBV152 (Figure 3B). Using binary regression, we assessed the time elapsed since vaccination and their association with development of a breakthrough infection and hospitalization, controlling for age (>60 years). Here, we showed that time elapsed since vaccination was an independent predictor of development of a breakthrough infection and hospitalization in those who had received the AZD1222 vaccine. As the level of anti-SARS-CoV-2 S1 IgG gradually decays with time, our regression model showed that each month of lapse was associated with increased risk of contracting a breakthrough infection and hospitalization by 0.85 (95% CI = 0.72–1.01; *P* = 0.048) and 0.85 (95% CI = 0.73–0.98; *P* = 0.041), respectively (Figure 3C).

**Table 3:** Factors associated with decay of anti-SARS-CoV-2 S1 IgG levels.

**Characteristics**	**Level of IgG, Coeff. (95% CI);** ***P*** **value**		
	**Natural infection**	**AZD1222^**†**^**	**BBV152^**‡**^**
Age, years	0.4 (−0.05 –0.85) *P* = 0.097	0.105 (−0.3 −0.61) *P* = 0.679	0.422 (−0.32–1.16) *P* = 0.264
Age (60), ≥60 years	14.5 (−7.22 –36.15) *P* = 0.391	14.44 (−8.14–37.02) *P* = 0.209	−8.18 (−45.9–29.55) *P* = 0.670
Gender, male	−5.3 (−18.8–8.65) *P* = 0.0470	0.52 (−15.06–16.1) *P* = 0.948	−4.51 (−27.25–18.23) *P* = 0.696
Comorbidities	6.98 (−12.57 – 26.53) *P* = 0.483	20.47 (−1.77 – 42.7) *P* = 0.71	−3.59 (−35.52–28.24) *P* = 0.825
Time elapsed since vaccination, month	…	– 5.31 (−9.17 to −1.44) ***P*** **=** 0.007**	−4.1 (−12.74 – 5.54) *P* = 0.351
Time since natural infection, month	−4.7 (−9.3 to −0.35) ***P*** **=** 0.034*	…	…
**Adjusted model**			
Time elapsed since vaccination, month	…	−6 (−9.88 to −2.1) ***P*** **=** 0.003**	−3.86 (−12.8–5.1) *P* = 0.576
Age (60), ≥ 60 years old		−23.32 (−46.69 to −0.05) ***P*** **=** 0.047*	4.68 (−38.2 −47.62) *P* = 0.83
Time elapsed since natural infection, month	−5.22 (−9.6 to −0.85) ***P*** **=** 0.019*	…	…
Age (60), ≥ 60 years old	20.7 (3.64–44.89) *P* = 0.195		

Factors associated with decay of anti-SARS-CoV-2 S1 IgG levels over time among study participants who experienced a natural infection vs. vaccination with the commercial AZD1222^†^ and BBV152^‡^. Since circulating IgG levels will decay gradually over time following a natural infection and/or vaccination, the levels of the antibodies were analyzed by using a linear regression model controlling for the days since participants become exposed to a natural infection or has completed the second dose of vaccination. Coeff., coefficient; CI, confident interval; month, define as 30 days. ^*^, ^**^, ^***^, ^****^, represent P-value < 0.05, <0.01, <0.001, <0.0001, respectively. The Hosmer–Lemeshow value for this model was P = 0.534.

*Included only age (60 years) in the adjusted model but not comorbidity. This is because old age and comorbidity are highly associated.^†^AZD1222 contains a replication-deficient chimpanzee adenovirus, which has a genetic material similar to that of SARS-CoV-2.^‡^BBV152 is a whole-virion inactivated vaccine*.

*Bold indicates significance*.

## Discussion

In this large population-based real-life investigation conducted in Chennai, Tamil Nadu, India, we studied 519 individuals examined for anti-SARS-CoV-2 S1 IgG antibody titers following either vaccination or recovery from documented COVID-19 infection. We investigated the factors associated with development of a breakthrough infection, as well as hospitalization, and correlated them with the dynamics of anti-SARS-CoV-2 S1 IgG titers, as well as factors that might be associated with the decay. We found that age and comorbid conditions were the two factors independently associated with development of a breakthrough infection and hospitalization in the study population. A combination of both age (>60 years) and underlying comorbid conditions were associated with increased risk for contracting a breakthrough infection and hospitalization by ~15 and ~10 times, respectively. Anti-SARS-CoV-2 S1 IgG decay was only observed among recipients of AZD1222, but not BBV152 and those who recovered from a natural SARS-CoV-2 infection. Due to the decay of anti-SARS-CoV-2 S1 IgG, we also reported that the risk of developing a breakthrough infection and hospitalization gradually increased by 0.85 times with each month.

It is pivotal to understand when and how a breakthrough infection with SARS-CoV-2 occurred in fully vaccinated individuals as it is paramount to determine how long the public health measures needs to be in place and whether or not a community required a booster dose (18). Immunity against viruses works primarily by inhibiting the infection phenomenon either by humoral (e.g., neutralizing antibodies) or by killing the infected cells *via* cell-mediated immune responses. While a vaccine works by generating immune memory in the form of memory B-cells and T-cells that permits a more rapid and intensified immune response against secondary infection; most vaccines are not completely designed to prevent exposure or transmission of an airborne pathogen such as SARS-CoV-2. Hence, acquisition of a breakthrough infection is determined by whether the vaccinated individual at the time of exposure has adequate levels of protective immunity to prevent the establishment of an infection (18). Many factors are known to influence immune surveillance including the age of the host, the dynamics of antibody responses (19), type/nature of vaccine used (4), interval between the vaccine doses (20, 21), underlying comorbid conditions and other health issues (viz., neoplasms and immunocompromised state) (22).

Several studies on the dynamics of anti-SARS-CoV-2 IgG levels post-vaccination and after recovering from a natural SARS-CoV-2 infection have revealed that the antibody levels induced by vaccines generally undergo rapid decay (over the months). One study reported that individuals who received a AZD1222 vaccine had a substantial decline in antibody levels after 6 months and that the decline was significantly associated with development of a breakthrough infection (23). Similarly, although individuals who received the BNT162b2 mRNA vaccine had high antibody titers compared to those who had survived a natural infection, the antibody titers experienced a rapid decay by up to 40% for every subsequent month; whereas the decrease of antibody levels was only <5% per month among convalescing individuals (24). This is consistent with our observation that individuals administered with AZD1222 experienced a more rapid attrition in IgG antibody levels.

B-cells that encounter their cognate antigens during an infection upon activation migrate to the center of the B-cell follicle, where they form germinal centers (GCs) (25, 26). Within the GCs, B-cells compete for a limited amount of T-cell-derived signals, such as cytokines and CD40 ligand that promote further maturation and differentiation into memory B-cells or plasmablasts (27, 28). Some of these plasmablasts will mature in the secondary lymphoid tissue itself into antibody-secreting plasma cells with short life spans; the other plasmablasts may enter into the circulation together with memory B-cells, home to bone marrow and other mucosal tissues, where they mature into long-lived plasma cells or memory B-cell that reside in these tissues to secrete antibodies for prolonged periods (25, 29, 30). Although both infection and vaccination can induce memory B-cells and plasmablasts that participate in humoral immune response, due to subtle differences in the nature of antigen stimulation, the memory B-cells generated in each case may be different. One study compared the memory B-cells induced following inoculation of a BNT162b2 mRNA vaccine and recovery from a natural infection and found that the mRNA vaccine induced robust plasmablast responses as compared to a natural infection that more prone to memory B-cells, thereby generating more plasma cells as well as better antigen-binding maturation (31). Another study compared the immune responses generated by both mRNA-based vaccine and the inactivated whole-virion vaccine and reported that the mRNA-based vaccine induced stronger humoral immune responses than the inactivated whole-virion vaccine (32). Further, the inactivated whole-virion vaccine induced significantly higher levels of IFN-γ response in CD4^+^ and CD8^+^ T cells as compared to that by the mRNA-based vaccine (32). Because T cell-derived signals (e.g., cytokines, ligands) are pivotal in promoting B cell maturation in germinal centers (27, 28), the inactivated whole-virion vaccine likely induced long-lived plasma cells and memory B cells.

Of note, there appears to be a fundamental difference between the adenovirus-based vaccine (AZD1222) and the mRNA-based subunit vaccine. Since adenovirus-based vaccine presents as a whole virus, it likely induces B-cell maturation much similar to inactivated SARS-CoV-2, and hence be able to generate long-lasting antibody responses. However, because this is an adenovirus-based vaccine and given that adenovirus is common in the population, the presence of anti-adenovirus neutralizing antibodies and anti-adenovirus specific T-cell response can prevent the vector from transducing the target cells, thereby limiting the efficacy of the vaccine (33). In fact, this likely could be a universal concern with all vaccines because of the presence of T and B cross-reactive memory responses to seasonal coronaviruses. Hence, it is difficult for a subunit vaccine that uses spike protein alone without adjuvants to induce long-lived plasma cells (34). These warrants improved vaccine formulations with suitable adjuvants to enhance antigenic stimulation.

Our study has also highlighted that the elderly age group (>60 years) and those with underlying comorbid conditions are at risk for acquisition of a breakthrough infection and hospitalization. Further, we have also reported that individuals administered with the AZD1222 also appear to undergo an accelerated decline in the levels of anti-SARS-CoV-2 S1 IgG. This likely could stem from an ongoing aging phenomenon involving the immune system known as immunosenescence (immune aging), where the generation of new T-cells appears to undergo progressive decline owing to thymic atrophy. The attrition is compensated for by the homeostatic proliferation of mature T-cells in the periphery. Eventually, the continually replicating mature T-cells undergo exhaustion due to telomere shortening (35) resulting in the expansion of senescent T-cell phenotypes indicated by the loss of co-stimulatory receptors (e.g. CD28, CD69) (36, 37), and de novo expression of co-inhibitory molecules such as killer-like immunoglobulin receptors (KIR) and PD-1 (35, 37). Our findings also reflect that the elderly with underlying comorbid conditions represent a high-risk population that requires additional medical attention, and specific measures to boost anti-SARS-CoV-2 immune responses (such as administering a second booster dose of the vaccines) in these groups are urgently warranted.

The limitation of this study is that we have not taken into account, the genetic variants of SARS-CoV-2 (38–41), especially the Delta and Omicron variants that have led to increased viral loads and high transmissibility. Notwithstanding, our study has provided a detailed information *via* a relatively large cohort involving both vaccinated and convalescent individuals recovering from SARS-CoV-2 infection. Our results indicate that the declining slope of anti-SARS-CoV-2 S1 IgG levels in AZD1222-vaccinated individuals is much steeper than in convalescent individuals and those who had received the BBV152 vaccine in Chennai, India. We have also provided an estimation of the rate of IgG decline as well as corresponding development of breakthrough infection and hospitalization risks by taking age, underlying comorbid conditions and time-scales into account. Given the expanding quantum of therapeutics against COVID-19, incapacitating the pandemic successfully is equally reliant on active public cooperation (42), which is of paramount importance to development of herd immunity against SARS-CoV-2.

## Data Availability Statement

The original contributions presented in the study are included in the article/supplementary material, further inquiries can be directed to the corresponding author.

## Ethics Statement

The studies involving human participants were reviewed and approved by Human Ethics Committee of the Madras Medical College (EC No. 03092021). The patients/participants provided their written informed consent to participate in this study.

## Author Contributions

The study was designed by SS, YY, GS, KN, SR, and ES. SS, GS, and KN provided regulatory oversight. SS and KN provided project management. SS, GS, KN, MR, and SR collected study data and oversaw participant visits. Participant data analysis and interpretation were done by YY, HT, YZ, ES, SR, and ML. Patient data collected and analyzed by SS, KN, GS, KV, PJ, DR, SP, SR, YY, HT, YZ, ES, SR, and ML and was interpreted by SS, KN, SR, YY, ML, and ES. SS, YY, HT, KN, GS, and ES wrote the manuscript. Data were accessed and verified by SS, GS, and SR. All authors contributed to the article and approved the submitted version.

## Conflict of Interest

The authors declare that the research was conducted in the absence of any commercial or financial relationships that could be construed as a potential conflict of interest.

## Publisher's Note

All claims expressed in this article are solely those of the authors and do not necessarily represent those of their affiliated organizations, or those of the publisher, the editors and the reviewers. Any product that may be evaluated in this article, or claim that may be made by its manufacturer, is not guaranteed or endorsed by the publisher.
